# Effects of Hypothermia vs Normothermia on Societal Participation and Cognitive Function at 6 Months in Survivors After Out-of-Hospital Cardiac Arrest: A Predefined Analysis of the TTM2 Randomized Clinical Trial

**DOI:** 10.1001/jamaneurol.2023.2536

**Published:** 2023-08-07

**Authors:** Gisela Lilja, Susann Ullén, Josef Dankiewicz, Hans Friberg, Helena Levin, Erik Blennow Nordström, Katarina Heimburg, Janus Christian Jakobsen, Marita Ahlqvist, Frances Bass, Jan Belohlavek, Roy Bjørkholt Olsen, Alain Cariou, Glenn Eastwood, Hans Rune Fanebust, Anders M. Grejs, Lisa Grimmer, Naomi E. Hammond, Jan Hovdenes, Juraj Hrecko, Manuela Iten, Henriette Johansen, Thomas R. Keeble, Hans Kirkegaard, Jean-Baptiste Lascarrou, Christoph Leithner, Mildred Eden Lesona, Anja Levis, Marco Mion, Marion Moseby-Knappe, Leanlove Navarra, Per Nordberg, Paolo Pelosi, Rachael Quayle, Christian Rylander, Helena Sandberg, Manoj Saxena, Claudia Schrag, Michal Siranec, Cassina Tiziano, Philippe Vignon, Pedro David Wendel-Garcia, Matt P. Wise, Kim Wright, Niklas Nielsen, Tobias Cronberg

**Affiliations:** 1 Clinical Studies Sweden, Forum South, Skane University Hospital, Lund, Sweden; 2 Cardiology, Department of Clinical Sciences Lund, Lund University, Skane University Hospital, Lund, Sweden; 3 Anesthesia and Intensive Care, Department of Clinical Sciences Lund, Lund University, Skane University Hospital, Malmö, Sweden; 4 Neurology, Department of Clinical Sciences Lund, Lund University, Skane University Hospital, Lund, Sweden; 5 Copenhagen Trial Unit, Center for Clinical Intervention Research, Copenhagen University Hospital, Copenhagen, Denmark; 6 Department of Regional Health Research, The Faculty of Health Sciences, University of Southern Denmark, Denmark; 7 Department of Anaesthesiology and Intensive Care Medicine, Institute of Clinical Sciences, Sahlgrenska Academy, University of Gothenburg, Sahlgrenska University Hospital, Gothenburg, Sweden; 8 Critical Care Program, The George Institute for Global Health and UNSW Sydney, Sydney, New South Wales, Australia; 9 Malcolm Fisher Department of Intensive Care, Royal North Shore Hospital, Sydney, New South Wales, Australia; 10 2nd Department of Medicine-Department of Cardiovascular Medicine, First Faculty of Medicine, Charles University in Prague and General University Hospital in Prague, Czech Republic; 11 Department of Anesthesiology, Sørlandet Hospital, Arendal, Norway; 12 Cochin University Hospital (APHP) and Paris Cité University (medical school), Paris, France; 13 Department of Intensive Care, Austin Hospital, Melbourne, Victoria, Australia; 14 Cardiac Intensive Care Unit, Haukeland University Hospital, Bergen, Norway; 15 Department of Intensive Care Medicine and Department of Clinical Medicine, Aarhus University Hospital, Aarhus, Denmark; 16 University Hospitals Bristol and Weston NHS Trust, Bristol, United Kingdom; 17 Department of Anesthesiology, Division of Emergencies and Critical Care, Oslo University Hospital, Rikshospitalet, Oslo, Norway; 18 The 1st Department of Internal Medicine, Cardioangiology, Medical Faculty of Charles University in Hradec Králové and University Hospital Hradec Králové, Hradec Králové, Czech Republic; 19 Department of Intensive Care Medicine, Inselspital, Bern University Hospital, University of Bern, Bern, Switzerland; 20 Department of Neurology, Rikshospitalet, Oslo University Hospital, Oslo, Norway; 21 Essex Cardio Thoracic Centre, Basildon, Essex, UK Thurrock University Hospitals, Basildon, United Kingdom; 22 MTRC, Anglia Ruskin University Faculty of Health Education Medicine & Social Care, Chelmsford, Essex, United Kingdom; 23 Research Center for Emergency Medicine, Emergency Department Aarhus University Hospital and Department of Clinical Medicine Aarhus University, Aarhus, Denmark; 24 Medecine Intensive Reanimation, CHU Nantes, Nantes, France; 25 Charité- Universitätsmedizin Berlin, coroporate member of Freie Universität Berlin and Humboldt- Universität-zu-Berlin, Department of Neurology, Berlin, Germany; 26 Intensive Care Unit, Wellington hospital, Wellington, New Zealand; 27 Department of Anesthesiology and Pain Medicine, Inselspital, Bern University Hospital, University of Bern, Bern, Switzerland; 28 Medical Research Institute of New Zealand, Wellington, New Zealand; 29 Center for Resuscitation Sciences, Department of Clinical Science and Education, Södersjukhuset, Karolinska Institutet, Stockholm, Sweden; 30 Function Perioperative Medicine and Intensive Care, Karolinska University Hospital, Stockholm, Sweden; 31 Department of Surgical Sciences and Integrated Diagnostics, University of Genoa, Genoa, Italy; 32 Anesthesia and Critical Care, San Martino Policlinico Hospital, IRCCS for Oncology and Neurosciences, Genoa, Italy; 33 Manchester Foundation Trust, Manchester, United Kingdom; 34 The Greater Manchester NIHR Clinical Research Network, Manchester, United Kingdom; 35 Department of Surgical Sciences, Anaesthesiology and Intensive Care Medicine, Uppsala University, Sweden; 36 Hallands hospital, Halmstad, Sweden; 37 St George Hospital Clinical School, The George institute for Global Health, University of New South Wales, Sydney, New South Wales, Australia; 38 Intensive Care Department, Kantonspital St Gallen, St Gallen, Switzerland; 39 Cardiac anesthesia and Intensive Care department, Istituto Cardiocentro Ticino, Lugano, Switzerland; 40 Medical-surgical ICU and Inserm CIC 1435, Dupuytren University hospital, Limoges, France; 41 Institute of Intensive Care Medicine, University Hospital Zurich, Zurich, Switzerland; 42 Adult Critical Care, University Hospital of Wales, Cardiff, United Kingdom; 43 Department of Clinical Sciences Lund, Anaesthesia and Intensive Care and Clinical Sciences Helsingborg, Helsingborg Hospital, Lund University, Lund, Sweden

## Abstract

**Importance:**

The Targeted Hypothermia vs Targeted Normothermia After Out-of-Hospital Cardiac Arrest (TTM2) trial reported no difference in mortality or poor functional outcome at 6 months after out-of-hospital cardiac arrest (OHCA). This predefined exploratory analysis provides more detailed estimation of brain dysfunction for the comparison of the 2 intervention regimens.

**Objectives:**

To investigate the effects of targeted hypothermia vs targeted normothermia on functional outcome with focus on societal participation and cognitive function in survivors 6 months after OHCA.

**Design, Setting, and Participants:**

This study is a predefined analysis of an international multicenter, randomized clinical trial that took place from November 2017 to January 2020 and included participants at 61 hospitals in 14 countries. A structured follow-up for survivors performed at 6 months was by masked outcome assessors. The last follow-up took place in October 2020. Participants included 1861 adult (older than 18 years) patients with OHCA who were comatose at hospital admission. At 6 months, 939 of 1861 were alive and invited to a follow-up, of which 103 of 939 declined or were missing.

**Interventions:**

Randomization 1:1 to temperature control with targeted hypothermia at 33 °C or targeted normothermia and early treatment of fever (37.8 **°**C or higher).

**Main outcomes and measures:**

Functional outcome focusing on societal participation assessed by the Glasgow Outcome Scale Extended ([GOSE] 1 to 8) and cognitive function assessed by the Montreal Cognitive Assessment ([MoCA] 0 to 30) and the Symbol Digit Modalities Test ([SDMT] z scores). Higher scores represent better outcomes.

**Results:**

At 6 months, 836 of 939 survivors with a mean age of 60 (SD, 13) (range, 18 to 88) years (700 of 836 male [84%]) participated in the follow-up. There were no differences between the 2 intervention groups in functional outcome focusing on societal participation (GOSE score, odds ratio, 0.91; 95% CI, 0.71-1.17; *P* = .46) or in cognitive function by MoCA (mean difference, 0.36; 95% CI,−0.33 to 1.05; *P* = .37) and SDMT (mean difference, 0.06; 95% CI,−0.16 to 0.27; *P* = .62). Limitations in societal participation (GOSE score less than 7) were common regardless of intervention (hypothermia, 178 of 415 [43%]; normothermia, 168 of 419 [40%]). Cognitive impairment was identified in 353 of 599 survivors (59%).

**Conclusions:**

In this predefined analysis of comatose patients after OHCA, hypothermia did not lead to better functional outcome assessed with a focus on societal participation and cognitive function than management with normothermia. At 6 months, many survivors had not regained their pre-arrest activities and roles, and mild cognitive dysfunction was common.

**Trial Registration:**

ClinicalTrials.gov Identifier: NCT02908308

## Key Points

**Question:**

Is there an effect of targeted hypothermia vs targeted normothermia on functional outcome focusing on societal participation and cognitive function in survivors 6 months after out-of-hospital cardiac arrest?

**Findings:**

In this predefined analysis of a randomized clinical trial, limitations in societal participation and cognitive impairment were common 6 months after out-of-hospital cardiac arrest with no differences between the 2 intervention groups. Younger survivors reported more limitations in societal participation.

**Meaning:**

In this study, targeted hypothermia had no significant effect on societal participation or cognitive function compared with targeted normothermia at 6 months in survivors of out-of-hospital cardiac arrest.

## Teaser

This study investigates the effects of targeted hypothermia vs targeted normothermia on functional outcome with focus on societal participation and cognitive function in survivors 6 months after out-of-hospital cardiac arrest.

## Introduction

Hypothermia was recommended in international guidelines as a neuroprotective strategy for those unconscious after out-of-hospital cardiac arrest (OHCA),^1,2,3^ but based on low certainty evidence.^2,4^ The Targeted Hypothermia vs Targeted Normothermia After Out-of-Hospital Cardiac Arrest (TTM2) trial^5^ reported no difference in mortality or poor functional outcome by the modified Rankin Scale (mRS) at 6 months after OHCA.^5^ A subsequent meta-analysis found no difference in 6-month mortality or functional outcome between temperature control with hypothermia (32 to 34 **°**C) and normothermia (36.5 to 38 **°**C).^6^ Guidelines for postresuscitation care were updated to recommend continuous monitoring of core temperature and active intervention to avoid fever (more than 37.7 **°**C) for at least 72 hours in comatose patients after cardiac arrest.^7,8^

The overall mortality in the TTM2 trial^5^ was 49% and 7% of survivors were dependent on others for daily activities corresponding to a poor functional outcome assessed by the mRS. While these results are consistent with previous literature, other studies using more detailed assessments have shown cognitive impairment to be common, affecting 30% to 50% of survivors of OHCA.^9^ Although classified as mostly mild or moderate, cognitive impairment may affect overall recovery and societal participation, such as return to work, leisure activities, and social relationships.^10^ The objective of this preplanned^11^ exploratory analysis of the TTM2 trial was to investigate the effects of hypothermia vs normothermia on functional outcome with a focus on societal participation and cognitive function in survivors 6-month after OHCA.

## Methods

### Design, Setting, and Participants

The randomized clinical TTM2 trial (NCT02908308)^11,12^ enrolled adult (18 years or older) unconscious patients with OHCA due to a presumed cardiac or unknown cause of arrest at 61 sites in 14 countries between November 2017 and January 2020. Participants were randomized less than 160 minutes after stable return of spontaneous circulation in a 1:1 ratio to temperature control with hypothermia at 33 **°**C or normothermia and early treatment of fever (temperature of 37.8 **°**C or higher).^12^ The randomization was stratified by site and coenrollment in the Targeted Therapeutic Mild Hypercapnia After Resuscitated Cardiac Arrest (TAME) trial.^5^ Hypothermia was maintained with a feedback-controlled device until 28 hours after randomization with rewarming at 1/3 **°**C per hour. A cooling device was used in the normothermia group if the core temperature reached 37.8 **°**C with the aim to keep the temperature at 37.5 **°**C or lower.^5^ A masked neurological prognostication was performed for all participants who remained in the intensive care unit at 96 hours after randomization or later, according to the protocol.^12^ All survivors were invited to a face-to-face follow-up at 6 months with a relative or close friend. For participants unable to attend face-to-face, parts of the follow-up were performed by telephone. If unable to participate in the follow-up, information from a proxy was used to assess outcome.^11^ The structured follow-up was performed according to the manual^13^ by local outcome assessors masked to the intervention. To increase interrater reliability, outcome assessors attended a national training meeting. To minimize avoidable missing data, a central coordinator (G.L.) provided support and reviewed the follow-up data at regular intervals.^11^ The last follow-up was performed on October 26, 2020. The primary and secondary outcomes of the TTM2 trial have been published.^5^ The Consolidated Standards of Reporting Trials Extension (CONSORT Extension) reporting guidelines were used when writing our report.^14^

### Consent

The TTM2 trial complies with the Declaration of Helsinki^15^ and the research protocol (Supplement 2) was approved by ethical committees in all participating countries. Written informed consent was obtained prior to the follow-up from all participants that regained mental capacity.

### Outcome Assessments

Descriptive characteristics were obtained at the time of randomization, during the hospital stay, and at the 6-month follow-up. The published protocol for outcome reporting in the TTM2 trial describes the rationale for the choice of outcome assessments and their psychometric properties.^11^

#### Societal Participation

The Glasgow Outcome Scale Extended (GOSE) score,^16^ a clinician-reported global functional outcome scale, including societal participation, was included. Information for the scoring was collected during an interview with the patient and/or a relative/proxy, and all available information.^17^ The GOSE categories range from 1 (dead) to 8 (upper level of good recovery) (eTable 1 in Supplement 1). A GOSE score less than 7 indicates limitations with societal participation. Return to work was used as a direct measure of societal participation, including occupational status prior to OHCA, at the time of the 6-month follow-up, and date of return to work.

#### Cognitive Function

The Montreal Cognitive Assessment (MoCA) version 7.1,^18^ a performance-based global cognitive screening measure with a total score range from 0 to 30 and scores less than 26 indicating cognitive impairment was used. The original MoCA requires a face-to-face meeting.^18^ In the telephone version (T-MoCA) the items visuoexecutive and naming are excluded, resulting in a total score range 0 to 22, with scores less than 19 indicating cognitive impairment.^19^ For both MoCA versions, participants with 12 years or less of education receive 1 additional point up to the maximum score. To enable analyses of the original MoCA and the T-MoCA combined, the T-MoCA was converted to a 30-item MoCA.^20^ When the combined version is used, this is here referred to as MoCA-30.

The Symbol Digit Modalities Test (SDMT),^21^ a performance-based assessment of mental processing speed and attention, was also used. SDMT raw scores (0 to 110) were transformed to age and education adjusted z scores for the oral and the written version separately. SDMT z scores were used for all analyses and z scores of −1 were used to indicate cognitive impairment.^18^ SDMT requires a face-to-face follow-up.

Descriptive information on subjective cognitive problems was assessed by the second question of the patient-reported Two Simple Questions (TSQ) survey^22,23^ asking “Do you feel that you have made a complete mental recovery from your heart arrest? (yes/no).” As an observer report (by a relative/close friend), the Informant Questionnaire on Cognitive Decline in the Elderly for Cardiac Arrest (IQCODE-CA) was used.^24,25^ The cutoff to capture changed cognitive performance in everyday life compared with before the cardiac arrest is above 3.04.^25^

### Statistical Methods

Analyses were preplanned and published, including power and sample size calculations.^11^ Potential differences between the 2 intervention groups were a priori limited to functional outcome with focus on societal participation (GOSE score), global cognition (MoCA), and mental processing speed/attention (SDMT).^11^ A comparison between the published protocol and the final analysis is presented in the eMethods in Supplement 1.

To avoid survival bias, the first analyses of the GOSE, MoCA-30, and SDMT include all participants, by using the full scale of the GOSE (scores 1 to 8) and by assigning deceased participants a lower score than the lowest possible for survivors for MoCA-30 and SDMT. These analyses were performed by the stratified Wilcoxon Mann-Whiney *U *test to account for site and coenrollment in the TAME trial.

For the second analysis including survivors only, a mixed-effects ordinal regression was used for GOSE (scores 2 to 8), presented as odds ratio (OR) for higher (better) scores for hypothermia compared with normothermia with 95% CIs. The model fulfilled the assumption of proportional odds. For MoCA-30 and SDMT, a mixed-effects linear regression was used. For all 3 outcomes, 2 separate models were performed. Model 1 includes adjustment for site (random intercept) and coenrollment in the TAME trial.^12^ Model 2 includes the same analyses but also adjustment for age (younger than 65 years and 65 years or older), education (university studies; yes or no), sex (male or female), and pre-arrest Clinical Frailty Scale score (1 to 4 and 5 to 9), if this was not already accounted for in the scoring, as age for SDMT, and education for MoCA-30 and SDMT.

Descriptive statistics for continuous data are presented as medians and interquartile ranges (IQRs) or means and SDs. Binary and categorical data are presented as numbers and percentages. All tests are 2-sided and a *P* value of <.05 indicates a statistically significant result. Results are considered exploratory and hypothesis generating only, with no adjustment for multiplicity. Statistical analyses were performed with R version 4.0.2 (The R Project).^26^

## Results

**Figure 1.**
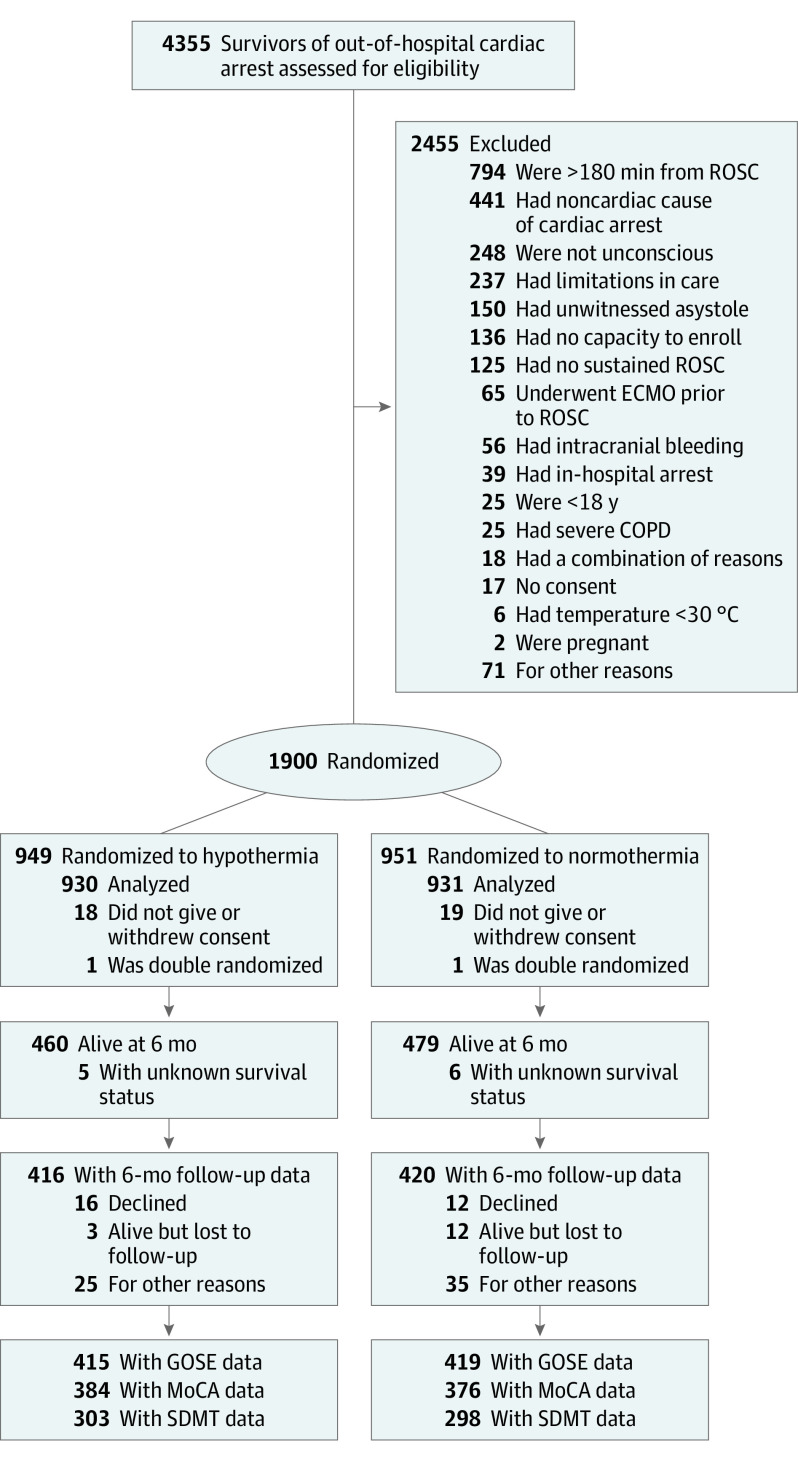
CONSORT Flow Diagram of Inclusion ECMO indicates extracorporeal membrane oxygenation; GOSE, Glasgow Outcome Scale Extended; MoCA, Montreal Cognitive Assessment; ROSC, return of spontaneous circulation; SDMT, Symbol Digit Modalities Test.

At 6 months, 939 of 1861 randomized participants were alive, (51%) of whom 836 participated in the structured follow-up (89%), with a similar distribution between the hypothermia and normothermia group (90% vs 88%). Face-to-face follow-up was performed in 619 of 836 of cases (74%). Some information on outcome was available for 82 of 103 participants who were alive but did not complete a structured follow-up (80%). Among them, 21 of 82 had a poor outcome based on all available information (26%) compared with 56 of 836 among those who completed the structured follow-up (7%). A CONSORT flow diagram is presented in Figure 1.

Characteristics pre-arrest, at the hospital, and at 6 months were similar between survivors in the 2 intervention groups participating in the follow-up (Table 1). At 6 months, most were living at home (403 in the hypothermia group [94%] and 384 in the normothermia group [97%]) and 121 in the hypothermia group (29%) and 111 in the normothermia group (26%) had attended cardiac rehabilitation, with only a few survivors having participated in neurorehabilitation (inpatient neurorehabilitation included 49 and 50 [12% both groups] and outpatient neurorehabilitation included 22 hypothermia [5%] and 29 normothermia [7%]) (Table 1).

**Table 1.** Patient Characteristics

Variable	Survivors participating in 6-mo follow-up	
	Hypothermia	Normothermia
No.	416	420
Age at time of cardiac arrest, mean (SD), y	60 (13)	59 (14)
Sex, No. (%)		
Male	354 (85)	346 (82)
Female	62 (15)	74 (18)
University-level education with or without degree, No. (%)	137 (33)	130 (32)
Medical history (pre-arrest), No. (%)		
Clinical Frailty Scale score 5-9	7 (2)	11 (3)
Charlson Comorbidity Index, median (IQR)	2 (1-3)	2 (1-3)
Poor functional outcome (mRS 4-5)	0 (0)	0 (0)
Memory problems (self-reported)	31 (8)	32 (8)
Myocardial infarction	53 (13)	62 (15)
Heart failure	23 (6)	27 (7)
Hypertension with pharmacological treatment	139 (35)	124 (31)
Diabetes	57 (14)	55 (13)
Prehospital resuscitation variables, No. (%)		
Location of cardiac arrest at home	175 (42)	181 (43)
Bystander-witnessed arrest	383 (92)	388 (92)
Bystander-performed cardiopulmonary resuscitation	353 (85)	359 (86)
First monitored rhythm shockable	371 (89)	380 (91)
Time (min) from OHCA to sustained ROSC, median (IQR)	20 (14-30)	20 (14-30)
Data on hospital admission, No. (%)		
Shock	84 (20)	85 (20)
FOUR motor score, median (IQR)	0 (0-0)	0 (0-0)
Bilaterally absent pupillary reflexes	286 (83)	289 (80)
In-hospital data		
Highest NSE value in ng/mL, median (IQR)^a^	26 (19-36)	22 (16-28)
CT diffuse and extensive anoxic brain injury^a^	7 (3)	7 (3)
MRI diffuse and extensive anoxic brain injury/all MRI^a^	3 (9)	3 (10)
Days in intensive care unit, median (IQR)	6 (4-9)	5 (3-9)
Days in hospital, median (IQR)	16 (11-25)	15 (10-24)
At time of 6-mo follow-up, No. (%)		
Days from cardiac arrest to follow-up, median (IQR)	186 (179-202)	187 (179-201)
Known neurological disease	33 (8)	27 (7)
Married/living as married	304 (73)	305 (75)
Living at home	403 (97)	384 (94)
Rehabilitation provided (self-reported), No. (%)		
Cardiac rehabilitation	121 (29)	111 (26)
Exercise-based cardiac rehabilitation	79 (19)	88 (21)
Inpatient neurological/cognitive/brain injury rehabilitation	49 (12)	50 (12)
Outpatient neurological/cognitive/brain injury rehabilitation	22 (5)	29 (7)
Other	24 (6)	18 (4)

Abbreviations: CT, computed tomography; ERC, European Resuscitation Council; ESICM, European Society of Intensive Care Medicine; FOUR, full outline of unresponsiveness; MRI, magnetic resonance imaging; mRS, modified Rankin Scale; NSE, neuron-specific enolase; OHCA, out-of-hospital cardiac arrest; ROSC, return of spontaneous circulation.

^a^ Missing data was frequent. NSE was based on the highest value at either 48 or 72 hours. The number included in these analyses were NSE, 416 of 420; CT, 246 of 261; and MRI, 29 of 33 for hypothermia and normothermia, respectively. The CT and MRI criteria for diffuse and extensive anoxic brain injury according to ERC/ESICM guidelines^3^ was based on information from a local radiologist only.

### Functional Outcome With Focus on Societal Participation

**Figure 2.**
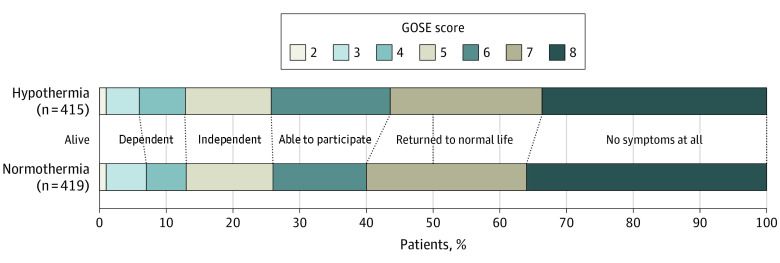
Functional Outcome Focusing on Societal Participation By the Glasgow Outcome Scale Extended (GOSE) score for survivors with hypothermia (n = 415) and normothermia (n = 419) at 6 months after out-of-hospital cardiac arrest. Information for the GOSE score was reported by the participant (328 of 415 vs 320 of 419), relative (11 of 415 vs 15 of 419), participant and relative together (72 of 415 vs 77 of 419), or other (3 of 415 vs 5 of 419). Description of categories included GOSE score of 2, vegetative state (unconscious); GOSE score of 3, lower severe disability (dependent, needs frequent help); GOSE score of 4, upper severe disability (dependent, needs some help); GOSE score of 5, lower moderate disability (independent, unable to participate in 1 or more life roles); GOSE score of 6, upper moderate disability (independent, limited to participate in 1 or more life roles); GOSE score of 7; lower good recovery (independent, returned to normal life with some symptoms); and GOSE score of 8, upper good recovery (independent and a full return to normal life).

The distribution of GOSE scores was similar between groups (Figure 2; eFigure 1 in Supplement 1) with a median of 7 (IQR, 5-8) for survivors in both groups and no differences between groups in the first analysis including deceased patients (n = 880 vs n = 865; *P* = .48) or in the second analyses of survivors only; first model (OR, 0.91; 95% CI, 0.71-1.17; *P* = .46 [n = 415 vs n = 419]) and second model with covariate adjustment (OR, 0.88; 95% CI, 0.68-1.13; *P* = .30 [n = 411 vs n = 404]). At 6 months, approximately one-third of survivors (Figure 2) in both groups had no symptoms at all (GOSE score of 8), while limitations with participation in 1 or more major life roles (GOSE score less than 7) were reported by 178 of 415 in the hypothermia group (43%) and 168 of 419 in the normothermia group (40%) (Figure 2). Younger OHCA survivors (younger than 65 years) reported more limitations in societal participation (GOSE score less than 7) compared with older OHCA survivors (65 years or older), 254 of 494 (50%) vs 101 of 340 (30%) (eFigure 2 in Supplement 1). GOSE scores for males and females were similar (eFigure 3 in Supplement 1).

Prior to the OHCA, 438 of 822 participants were working (53%) (eTable 2 in Supplement 1). At 6 months, half of the participants who were working pre-arrest (219 of 438 [50%]) had returned to their previous (or higher) level of work. The rate of return to work was similar between the hypothermia and normothermia groups (eTable 2 in Supplement 1). When including those with an adjustment to fewer hours of work, the number that had returned to work increased to 63% (275 of 438). The median time to return to work was 80 (IQR, 46-112) days from the OHCA. Most that had not returned to work were on sick leave.

### Cognitive Function

Global cognitive function by MoCA was assessed for 760 of 939 survivors (81%) and in 607 of 760 by the original face-to-face version (80%). There were no differences between the groups by the MoCA-30, neither in the first analyses including deceased nor in analyses of survivors only (Table 2). While the median MoCA-30 score was within the normal range for both the hypothermia (27; IQR, 23-29) and the normothermia group (26; IQR, 23-28), 149 of 384 in the hypothermia group (39%) and 160 of 376 in the normothermia group (43%) had MoCA-30 scores below the cutoff indicating cognitive impairment (Table 3). The most affected MoCA-30 items were verbal fluency and delayed recall (eTable 3 in Supplement 1). Results were similar between MoCA performed face-to-face and T-MoCA (Table 3; eTable 3 in Supplement 1).

**Table 2.** Secondary Analyses of Cognitive Function for Out-of-Hospital Cardiac Arrest Survivors With Hypothermia vs Normothermia^a^

Outcome assessment^b^	All including dead; *P* value	Model 1: survivors at 6 mo only		Model 2: survivors at 6 mo with adjustments for clinical characteristics	
		Mean difference (95% CI)	*P* value	Mean difference (95% CI)	*P* value
MoCA-30^c^	.88	0.36 (−0.33 to 1.05)	.37	0.38 (−0.29 to 1.05)	.27
SDMT z score	.82	0.06 (−0.16 to 0.27)	.62	0.03 (−0.19 to 0.25)	.77

Abbreviations: MoCA, Montreal Cognitive Assessment; SDMT, Symbol Digit Modalities Test.

^a^ Performed by mixed-effects linear regression: model 1, adjustment for site (random intercept) and coenrollment in the TAME trial, model 2 also including adjustment for age (younger than 65 years and 65 years or older; MoCA only), sex (male or female), and pre-arrest Clinical Frailty Score (1 to 4 and 5 to 9).

^b^ Number of outcome assessments (hypothermia or normothermia), MoCA-30, including dead (849 of 930 vs 822 of 931) and including survivors only (384 of 460 vs 376 of 479), and SDMT including dead (768 of 930 vs 744 of 931) and including survivors only (303 of 460 vs 298 of 479).

^c^ Including converted T-MoCA.

**Table 3.** Outcome Assessment of Cognitive Function at 6-Month Follow-Up

Outcome assessment	Survivors with 6 mo follow-up	
	Hypothermia	Normothermia
No.	460	479
MoCA, No.	305	302
MoCA, median (IQR)	27 (24-29)	26 (23-28)
MoCA <26, No. (%)	117 (38)	131 (43)
T-MoCA, No.	79	74
T-MoCA, median (IQR)	19 (17-21)	19 (17-21)
T-MoCA <19, No. (%)	32 (41)	29 (39)
MoCA-30, No.^a^	384	376
MoCA-30, median (IQR)^a^	27 (23-29)	26 (23-28)
MoCA-30, mean (SD)^a^	25 (5)	25 (5)
MoCA-30 <26, No. (%)^a^	149 (39)	160 (43)
SDMT, No.	303	298
SDMT z score, median (IQR)	−0.91 (−1.78 to −0.12)	−0.96 (−1.96 to −0.18)
SDMT z score, mean (SD)	−1.01 (1.40)	−1.09 (1.36)
SDMT<−1 SD, No. (%)	142 (47)	144 (48)
SDMT<−1.5 SD, No. (%)	90 (30)	103 (35)
TSQ, No.	412	404
TSQ question 2 = no	140 (34)	139 (34)
IQCODE-CA, No.	365	364
IQCODE-CA, median (IQR)	3.00 (3.00-3.10)	3.00 (3.00-3.02)
IQCODE-CA >3.04, No. (%)	126 (35)	135 (37)
IQCODE-CA by informant living with the patient, No. (%)	263 (72)	267 (73)

Abbreviations: IQCODE-CA, Informant Questionnaire on Cognitive Decline in the Elderly for Cardiac Arrest; MoCA, Montreal Cognitive Assessment; SDMT, Symbol Digit Modalities Test; T-MoCA, Telephone version of the Montreal Cognitive Assessment; TSQ, Two Simple Questions.

^a^ Including converted T-MoCA.

Mental processing speed/attention by the SDMT was assessed for 601 of 939 participants (64%). There were no differences between groups for SDMT in any of the analyses (Table 2), with a median SDMT z score of −0.91 (IQR, −1.78 [−0.12]) for the hypothermia group and −0.96 (IQR, −1.96 [−0.18]) for the normothermia group. Almost half in both groups (hypothermia, 142 of 303 [47%] vs normothermia, 144 of 298 [48%]), had SDMT scores indicating cognitive impairment (Table 3).

Among the participants who performed both MoCA-30 and SDMT, 353 of 599 had scores indicating cognitive impairment in at least 1 of the assessments (59%) and nearly one-third (176 of 599 [29%]) had scores indicating impairment on both assessments. A total of 108 of 599 had low scores on SDMT only (18%) and 69 of 599 on MoCA-30 only (11%).

Patient-reported problems with mental recovery assessed by TSQ were reported by 140 in the hypothermia group and 139 in the normothermia group (34% in both). In the observer-reported IQCODE-CA assessment, cognitive problems were similar between groups, 126 in the hypothermia group (35%), and 135 in the normothermia group (37%) (Table 3).

## Discussion

In this preplanned study of the TTM2 trial, we found that hypothermia compared with normothermia did not affect functional outcome focusing on societal participation or cognitive function in survivors at 6 months. One-third of participants had no symptoms at all; however, 40% reported impairment in a major life domain and mild cognitive impairment was common.

In resuscitation science, functional outcome is often dichotomized as good or poor, closely reflecting survival status as few participants survive with severely impaired function. Although relevant to the practice of withdrawal of life-sustaining therapies after neurological prognostication, dichotomized good outcome may still include survivors with significant problems.^9^ Dichotomizing outcomes decreases the ability to identify small but possibly patient-important effects and the long-term impact of interventions on health of cardiac arrest survivors and their families may be underestimated.

To capture the consequences on societal participation for survivors of OHCA, we used the GOSE. GOSE is similar to the mRS, the currently recommended scale for functional outcome after cardiac arrest,^27^ and used as a secondary outcome in the TTM2 trial.^5^ Compared with mRS, the GOSE provides more details regarding societal participation and role functioning. GOSE scoring is also supported by a structured interview^16^ and a published manual.^17^ The dichotomized level of good functional outcome in relation to independence in basic activities of daily living for survivors was similar in this trial by mRS (0 to 3) and GOSE (4 to 8), 93% vs 94%.

Although most survivors of OHCA were living at home and considered to have an overall good functional outcome, 346 of 834 participants (42%) reported at least some limitations with participation in normal activities and roles, similar between the 2 temperature groups. A greater proportion of younger survivors reported limitations with societal participation (GOSE score 5 to 6) while still being independent in daily activities. A previous study^28^ reported more affective and cognitive sequelae in younger survivors of OHCA.^28^ These findings may be related to higher percentage of survival^29^ or increased demands of everyday life among younger survivors of OHCA.^17^ In agreement with this, half of pre-arrest workers in our study had not returned to their previous level of work at 6 months. Ability to work is associated with health and well-being^30^ and inability to work has important financial consequences for survivors, their families, and the society.^31^

We found cognitive dysfunction to be common 6 months after OHCA. This finding has been previously reported,^32^ but a recent review questioned the generalizability of these results.^32^ We present data from a large sample of survivors of OHCA, assessed by a standard protocol in multiple sites and few missing data. Given the nonexistent differences between temperatures, this cohort represents robust data for survivors of OHCA managed at different target temperatures at 6 months.

MoCA is recommended for cognitive screening after cardiac arrest.^3,33^ We found that MoCA was also well accepted by participants and outcome assessors with more than 90% of follow-up participants having an assessment, which demonstrated the feasibility of using MoCA after OHCA. T-MoCA is an alternative to avoid missing data when face-to-face testing is not possible.^19^ The psychometric properties for the T-MoCA are sufficient, but some of the most discriminative items are excluded^18^ and the sensitivity is therefore lower.^19,20^ In this study, classification by the face-to-face MoCA and the T-MoCA was similar, but the validity of the T-MoCA for survivors of OHCA needs further evaluation.

Although the sensitivity for MoCA is relatively high, this may be further increased by adding an assessment of processing speed, such as the SDMT.^34,35^ The 2 assessments in combination identified potential problems in more than half of survivors and the most affected domains were executive function (MoCA; verbal fluency), memory (MoCA; delayed recall), and processing speed/attention (SDMT). This pattern of cognitive impairment has been reported previously in survivors of cardiac arrest by detailed, but lengthy, neuropsychological assessments.^32^ Importantly, the high sensitivity desired for cognitive screening for the MoCA comes with a lower specificity and for SDMT the cutoff for cognitive impairment in this trial includes 16% of the normal population. To avoid overestimations, those identified with a cognitive-screening instrument should be further evaluated by someone experienced in cognitive assessments and in relation to premorbid function and consequences for daily life.

Including survivors’ and their families’ perspective on outcome is recommended.^36^ New cognitive problems in daily life as reported by the participants (TSQ) and the informants (IQCODE-CA) occurred in 34% to 37% of cases, which is less compared with MoCA and SDMT. That objective and subjective outcomes do not necessarily overlap has been reported and may be due to several factors.^37,38^ Cognitive problems may also be related to for example, preexisting vascular brain damage, age, or psychological stress.^9^

Cognitive impairment is a risk factor for reduced societal participation and return to work after cardiac arrest.^10,30^ There are, however, other factors that may be important, such as depression,^10^ mobility problems,^10^ fatigue,^10,30^ restrictions (due to implantable cardioverter-defibrillator or medications),^30^ type of work,^30,39^ national differences,^10^ and provision of support.^30^

Even if most neurological recovery occurs during the first months after OHCA, there may be functional improvements later. A large registry-based Danish study^40^ showed a median time for return to work of 4 months but with a large variation (IQR, 1 to 19 months).^40^ Peskine et al^41^ reported continuing improvement for behavioral disabilities and health-related quality of life up to 12 months post-OHCA. The topic of late recovery will be further investigated in the TTM2 trial using data from an additional follow-up more than 24 months post-OHCA.

### Limitations

Due to the COVID-19 pandemic, the number of face-to-face follow-ups decreased and was lower compared with the previous TTM trial (74% vs 92%),^42^ primarily affecting SDMT assessments which require a physical visit. As the sample size was based on the primary outcome (survival),^11^ the power for these analyses is still assumed sufficient and all differences were smaller than prespecified levels of clinically relevant effect sizes.^11^ Participants missing from the structured follow-up were more likely to have a poor outcome, but because they constituted a small number, they likely did not have important effects on the overall results. We cannot conclude on any rehabilitation effect, but overall, a low number had participated in rehabilitation, especially neurological rehabilitation. Another limitation is that we lack information on the confounding factors mood and behavioral dysfunction. Lastly, the results of this study may not be generalizable to all cardiac arrest populations since we only included survivors from OHCA of cardiac or unknown cause.

## Conclusions

In this predefined analysis of comatose survivors after OHCA, hypothermia did not lead to better societal participation or cognitive function outcomes than management with normothermia. The implication of this study is the addition of more evidence that hypothermia is not clinically beneficial as compared with maintaining normothermia.

## Notes

Supplement 1. eMethods. Institutions where the work was performed, comparison to the study protocol, and acknowledgement and study organization eTable 1. Detailed description of the categories in the Glasgow Outcome Scale Extended (GOSE) eTable 2. Occupational status at time of, and at 6-months after Out-of-hospital cardiac arrest eTable 3. Results of individual items of the Montreal Cognitive Assessment (MoCA) eFigure 1. Functional outcome focusing on societal all participants including death eFigure 2. Functional outcome focusing on societal participation by age groups eFigure 3. Functional outcome focusing on societal participation by sex

Supplement 2. Original protocol TTM2 trial

Supplement 3. Data sharing statement
