# Supplementary material for: Effects of Hypothermia vs Normothermia on Societal Participation and Cognitive Function at 6 Months in Survivors After Out-of-Hospital Cardiac Arrest: A Predefined Analysis of the TTM2 Randomized Clinical Trial
Source: JAMA Neurol. 2023 Aug 7;80(10):1070–9. doi: 10.1001/jamaneurol.2023.2536 (PMC10407762; doi:10.1001/jamaneurol.2023.2536)
Supplement: Supplement 1. — eMethods. Institutions where the work was performed, comparison to the study protocol, and acknowledgement and study organization eTable 1. Detailed description of the categories in the Glasgow Outcome Scale Extended (GOSE) eTable 2. Occupational status at time of, and at 6-months after Out-of-hospital cardiac arrest eTable 3. Results of individual items of the Montreal Cognitive Assessment (MoCA) eFigure 1. Functional outcome focusing on societal all participants including death eFigure 2. Functional outcome focusing on societal participation by age groups eFigure 3. Functional outcome focusing on societal participation by sex [file jamaneurol-e232536-s001.pdf]

Supplemental Online Content

Lilja G, Ullén S, Dankiewicz J, et al. The effects of hypothermia vs normothermia on societal participation and cognitive function at 6 months in survivors after out-of-hospital cardiac arrest: a predefined analysis of the randomized clinical TTM2 randomized clinical trial. *JAMA Neurol*. Published online August 7, 2023.
doi:10.1001/jamaneurol.2023.2536

eMethods. Institutions where the work was performed, comparison to the study protocol, and acknowledgement and study organization
eTable 1. Detailed description of the categories in the Glasgow Outcome Scale Extended (GOSE)
eTable 2. Occupational status at time of, and at 6-months after Out-of-hospital cardiac arrest
eTable 3. Results of individual items of the Montreal Cognitive Assessment (MoCA)
eFigure 1. Functional outcome focusing on societal all participants including death
eFigure 2. Functional outcome focusing on societal participation by age groups
eFigure 3. Functional outcome focusing on societal participation by sex

This supplemental material has been provided by the authors to give readers additional information about their work.

© 2023 American Medical Association. All rights reserved.

eMethods. Institutions where the work was performed

Australia: Royal North Shore Hospital, Sydney; Concord Repatriation General Hospital, Sydney; The Alfred Hospital, Melbourne; Nepean Hospital, Sydney; Northern Hospital, Melbourne; Austin Hospital, Melbourne; St. Vincent's Hospital, Sydney; Princess Alexandra Hospital, Brisbane; John Hunter Hospital, Newcastle; Liverpool Hospital, Sydney. **Austria:** Medical University Innsbruck, Innsbruck. **Belgium:** Erasme University Hospital, Brussels; Ziekenhuis Oost-Limburg, Genk. **Czech Republic:** General University Hospital, Prague; University Hospital Hradec Králové, Hradec Králové; Regional Hospital Liberec, Liberec. **Denmark:** Aarhus University Hospital, Aarhus. **France:** Cochin University Hospital (APHP), Paris; Lariboisiere University Hospital (APHP), Paris; Centre Hospitalier deVersailles, Le Chesnay; CHU de Nantes, Nantes; Dupuytren Teaching Hospital, Limoges. **Germany:** Charitè Unviersity Hospital, Berlin. **Italy:** San Martino Policlinico Hospital, Genoa; Civil Hospital, Baggiovara, Modena; Trieste University Hospital, Trieste. **New Zealand:** Wellington Regional Hospital, Wellington; Christchurch Hospital, Christchurch. **Norway:** Oslo University Hospital, Rikshospitalet, Oslo; St Olav's University Hospital, Trondheim; Sorlandet Hospital, Arendal; Haukeland University Hospital, Bergen. **Sweden:** Skåne University Hospital, Malmö; Skåne University Hospital, Lund; Helsingborg Hospital, Helsingborg; Södersjukhuset, Karolinska Institute, Stockholm; Capio Sankt Göran Hospital, Stockholm; Sahlgrenska University Hospital, Göteborg; Uppsala University Hospital, Uppsala; Linköping University Hospital, Linköping; Northern Älvsborg County Hospital, Trollhättan; Skaraborg Hospital, Skövde; Örebro University Hospital, Örebro; Halland hospital, Halmstad; Karlstad Central Hospital, Karlstad. **Switzerland:** Lausanne University Hospital, Lausanne; Bern University Hospital, Bern; Cardiocentro Ticino, Lugano; Cantonal Hospital St. Gallen, St Gallen; University Hospital Zurich, Zurich. **United Kingdom:** University Hospital of Wales, Cardiff; Royal Victoria Hospital, Belfast; Essex Cardiothoracic Centre, Basildon; Royal Berkshire Hospital, Reading; Manchester Royal Infirmary, Manchester; Bristol Royal Infirmary, Bristol; University Hospital Birmingham NHS foundation Trust, Birmingham; Queen Alexandra Hospital, Portsmouth; Royal Bournemouth Hospital, Bournemouth. **USA:** University of Pittsburgh, Pittsburgh; Mayo Clinic, Rochester.

Comparison to the study protocol

Minor modifications from the published protocol for outcome reporting and follow-up in the Targeted Hypothermia versus Targeted Normothermia after Out-of-hospital Cardiac Arrest trial (TTM2) were made: Firstly, we planned to use SDMT raw scores (0-110) from the Symbol Digit Modalities Test to asses mental processing speed/attention. As both oral and written versions of the test were used to a large extent in the trial,

© 2023 American Medical Association. All rights reserved.

and since the two versions have different normative values, it was considered more appropriate to use age and education adjusted z-scores for all analyses. Therefore, age and education were not included in the co-variate adjusted analysis, as adjustment was already included in the test score.
According to protocol the first analyses of MoCA and SDMT include deceased in the non-parametric van Elteren test. However, as this test was not available in the R: language and environment for statistical computing, the stratified Wilcoxon Mann Whitney U test was used instead for these analyses.

Acknowledgement and study organization

TTM2 collaborators:

STEERING GROUP

Niklas Nielsen, Lund University, Helsingborg Hospital, Department of Clinical Sciences Lund, Anesthesiology and Intensive care, Lund, Sweden (Chair and Chief Investigator); Jan Bělohlávek, 2nd Department of Medicine, First Faculty of Medicine, Charles University in Prague and General University Hospital, Prague, Czech Republic (NI); Clifton Callaway, Department of Emergency Medicine, University of Pittsburgh, Pittsburgh, PA, USA (NI); Alain Cariou, Descaretes University of Paris and Cochin University Hospital, Paris, France (NI); Tobias Cronberg, Lund University, Skåne University Hospital Lund, Department of Clinical Sciences, Neurology, Lund, Sweden (Senior Investigator); Josef Dankiewicz, Lund University, Skåne University Hospital Lund, Department of Clinical Sciences, Cardiology, Lund, Sweden (Coordinating Investigator); Glenn Eastwood, The Australian and New Zealand Intensive Care Research Centre, Monash University, Melbourne, Australia; David Erlinge, Lund University, Skåne University Hospital Lund, Department of Clinical Sciences, Cardiology, Lund, Sweden; Hans Friberg, Lund University, Skåne University Hospital Malmö, Department of Clinical Sciences, Anesthesia & Intensive care, Lund, Sweden (Senior Investigator); Jan Hovdenes, Department of Anesthesiology and Intensive Care, Oslo University Hospital, Rikshospitalet, Oslo, Norway (NI); Janus Christian Jakobsen, Copenhagen Trial Unit, Capital Region, Copenhagen, Denmark; Department of Regional Health Research, The Faculty of Health sciences, University of Southern Denmark, Denmark (Trialist); Michael Joannidis, Division of Intensive and Emergency Medicine, Department of Internal Medicine, Medical University Innsbruck, Innsbruck, Austria (NI); Hans Kirkegaard, Research Center for Emergency Medicine, Department of Clinical Medicine, Aarhus University Hospital and Aarhus University, Aarhus N, Denmark (NI); Helena Levin, Lund University, Skåne University Hospital Lund, Department of Clinical Sciences, Anesthesiology and Intensive care, Lund, Sweden (Clinical Trial Manager); Gisela Lilja, Lund University, Skåne University Hospital Lund, Department of Clinical Sciences,

© 2023 American Medical Association. All rights reserved.

Neurology, Lund, Sweden (Follow-up Coordinator); Matt P. G. Morgan, Adult Critical Care, University Hospital of Wales, Cardiff, United Kingdom; Alistair D. Nichol, University College Dublin- Clinical Research Centre at St Vincent's University Hospital, Dublin, Ireland. Per Nordberg, Department of Medicine, Center for Resuscitation Science, Karolinska Institute, Solna, Sweden; Mauro Oddo, Neuroscience Critical Care Group, Adult Intensive Care Medicine Service, CHUV-Lausanne University Hospital, University of Lausanne, Lausanne, Switzerland (NI); Paolo Pelosi, Anesthesiology and Critical Care, San Martino Policlinico Hospital, IRCCS for Oncology and Neurosciences, Department of Surgical Sciences and Integrated Diagnostics, University of Genoa, Genoa, Italy (NI); Christian Rylander, Department of Anesthesiology and Intensive Care Medicine, Institute of Clinical Sciences, Sahlgrenska Academy, University of Gothenburg, Gothenburg, Sweden (NI); Manoj Saxena, Division of Critical Care and Trauma, George Institute for Global Health. Bankstown-Lidcombe Hospital, South Western Sydney Local Health District, Sydney, Australia (NI); Christian Storm, Department of Nephrology and Medical Intensive Care, Charité - Universitätsmedizin Berlin, Germany (NI); Fabio S. Taccone, Department of Intensive Care, Erasme University Hospital, Université Libre de Bruxelles (ULB), Brussels, Belgium (NI); Susann Ullén, Clinical Studies Sweden – Forum South, Skåne University Hospital, Lund, Sweden (Chief Statistician); Matt P. Wise, Adult Critical Care, University Hospital of Wales, Cardiff, United Kingdom (NI); Paul J. Young, Medical Research Institute of New Zealand, Intensive Care Unit, Wellington Hospital, Wellington, New Zealand (NI).

NI- National Coordinating Investigator

INDEPENDENT DATA MONITORING AND SAFETY COMMITTEE

Kathy Rowan, Intensive Care National Audit & Research Centre, UK (Chair); David Harrison, Intensive Care National Audit & Research Centre, UK; Paul Mouncey, Intensive Care National Audit & Research Centre, UK; Manu Shankar-Hari, Guy’s and St Thomas’s NHS Foundation Trust, London, UK; Duncan Young, Nuffield Department of Clinical Neurosciences, University of Oxford, UK.

STATISTICIANS

Susann Ullén, Clinical Studies Sweden – Forum South, Skåne University Hospital, Lund, Sweden (Chief Statistician); Theis Lange, Department of Biostatistics, University of Copenhagen, Copenhagen, Denmark (Independent Statistician); Karolina Palmér, Department of Medical Statistics and Epidemiology, Region Skåne, Malmö, Sweden (Independent statistician).

COORDINATING ORGANIZATIONS AND TRIAL MANAGEMENT

Region Skåne, Helsingborg Hospital, Helsingborg, Sweden (Sponsor)

Lund University, Lund, Sweden

© 2023 American Medical Association. All rights reserved.

Core management group: Niklas Nielsen (Chair and Chief Investigator), Josef Dankiewicz (Coordinating Investigator), Tobias Cronberg (Senior Investigator, Neurology), Hans Friberg (Senior Investigator, Intensive Care), Gisela Lilja (Follow-up Coordinator), Helena Levin

(Clinical Trial Manager), Janus Christian Jakobsen (Trialist), Susann Ullén (Chief Statistician).

Trial financial management: Helsingborg Hospital: Ulla-Britt Karlsson; Lund University: Simon Heissler.

Australia: The George Institute for Global Health, Sydney (Local Sponsor):

Manoj Saxena, Frances Bass, Naomi Hammond, John Myburgh, Colman Taylor.

France: Clinical Research Unit, Paris Descartes Necker Cochin, Paris (Local Representative): Alain Cariou, Adele Bellino.

TRIAL COORDINATORS AND MONITORS

Australia: The George Institute for Global Health, Sydney: Marwa Abel-all, Ben Finfer, Carolyn Koch, Yang Li, Anne O'Connor, Julia Pilowsky, Tina Schneider, Anna Tippett; Monash University, Melbourne: Bridget Ady, Tessa Broadley, Amanda Brown, Liz Melgaard, Mimi Morgan, Vanessa Singh, Rebecca Symons. **Austria:** Medical University, Innsbruck, Innsbruck: Kathrin Becker. **Belgium:** NVS Consulting, Brussels: Nathalie Van Sante. Czech Republic: Aixial, Brno: Vendula Saleova, Silvie Zerzanova. **Denmark:** Lund University, Lund, Sweden: Helena Levin. **France:** Clinical Research Unit, Paris Descartes Necker Cochin, Paris: Samia Sefir-Kribel. **Germany:** Charité Universitätsmedizin, Berlin: Ute Lübeck. Italy: Mario Negri Institute for Pharmacological Research, Milan: Martina Carrara. **New Zealand:** Medical Research Institute of New Zealand (MRINZ), Wellington: Kathryn Fernando, Diane Mackle, Leanlove Navarra, Judith Riley. **Norway:** Oslo University Hospital, Oslo: Elin Westerheim; Haukeland University Hospital, Bergen: Marianne Flatebø. **Sweden:** Helsingborg Hospital, Helsingborg: Ameldina Ceric, Zana Haxhija, Lovisa Terling; Skåne University Hospital, Lund: Lena Bossmar, Liz Jergle, Helén Holm Månsson. **Switzerland:** Lausanne University Hospital (CHUV), Lausanne: Samia Abed Maillard, Andreja Vujicic Zagar; Cantonal Hospital St. Gallen, St. Gallen: Christina Jodlauk. **United Kingdom:** University Hospital of Wales, Cardiff: Helen Hill; Niche Science & Technology, Richmond: Jennifer Scrivens; The HRB Irish Critical Care- Clinical Trials Network (ICC-CTN), Dublin, Ireland: Kate Ainscough, Ciara Fahey.

SITES, PRINCIPAL INVESTIGATORS, AND SITE PERSONNEL

Australia: Austin Hospital, Melbourne: Rinaldo Bellomo (PI), Glenn Eastwood, Leah Peck, Helen Young; Concord Repatriation General Hospital, Sydney: Winston Cheung (PI), Rosalba Cross, Michael Hayes, Nitin Jain, Mark Kol, Asim Shah, Atul Wagh, Helen Wong; John Hunter Hospital, Newcastle: F. Eduardo Martinez (PI), Gail

© 2023 American Medical Association. All rights reserved.

Brinkerhoff, Dustin Bush; Liverpool Hospital, Sydney: Antony Stewart (PI), Anders Aneman, Lien Lombardo, Peter McCanny, James Penketh; Nepean Hospital, Sydney: Ian Seppelt (PI), Rebecca Gresham, Julie Lowrey, Kristy Masters, Christina Whitehead; Princess Alexandra Hospital, Brisbane: James Walsham (PI), Meg Harward, Josephine Mackay, Jason Meyer, Emma Saylor, Ellen Venz, Krista Wetzig; Royal North Shore Hospital, Sydney: Wade Stedman (PI), Angela Ashelford, Frances Bass, Naomi Hammond, Sharon Mar, Julia Pilowsky, Miyuki Tokumitsu, Elizabeth Yarad; St Vincent's Hospital, Sydney: Hergen Buscher (PI), Claire Reynolds; The Alfred Hospital, Melbourne: Andrew Udy (PI), Aidan Burrell, Jasmin Collins, Dashiell Gantner, Victoria Emma-Leah Martin, Phoebe Mccracken, Vinodh Nanjayya, AlistairNichol, Alexander Sacha Richardson, Meredith Young; Northern Hospital, Melbourne: Angaj Ghosh (PI), Simone Said.

Austria: Medical University Innsbruck, Innsbruck: Michael Joannidis (PI), Ronny Beer, Frank Hartig, Raimund Helbok, Sebastian Klein, Andreas Peer.

Belgium: Erasme University Hospital, Brussels: Fabio S. Taccone (PI), Jacques Creteur, Dominique Durand; Ziekenhuis Oost-Limburg, Genk: Matthias Dupont (PI), Sigrid Christiaens, Carola Claes, Sebastiaan Deckx, Bert Ferdinande, Sanne Lenaerts, Wilifred Mullens, Sarah Stroobants, Evi Theunissen, David Verhaert.

Czech Republic: General University Hospital, Prague: Ondřej Šmíd (PI), Marek Flaksa, David Kemlink, Jan Malík, Michal Otáhal, Jan Rulíšek, Michal Šíranec, Zdeněk Stach, Anna Valeriánová, Petra Zavadilova; University Hospital Hradec Králové, Hradec Králové: Miroslav Solař (PI), Róber Bánszky, Jana Červená, Renata Černá Pařízková, Libor Šimůnek, Filip Varhaník; Regional Hospital Liberec, Liberec: Jiří Karásek (PI), Matěj Strýček.

Denmark: Aarhus University Hospital, Aarhus: Anders Grejs (PI), Steffen Christensen, Peter Juhl-Olsen, Ida Katrine Thomsen, Lisa Gregersen Østergaard.

France: Cochin University Hospital (APHP), Paris: Alain Cariou (PI), Albert Cao, Pierre Dupland, Ariane Gavaud, Paul Jaubert, Mathieu Jozwiak, Nathalie Marin, Guillaume Savary; Lariboisiere University Hospital (APHP), Paris: Nicolas Deye (PI), Bruno Megarbane, Pierre Mora, Laetitia Sutterlin; Centre Hospitalier de Versailles, Le Chesnay: Stephane Legriel (PI), Hugo Bellut, Alexis Ferre, Guillaume Lacave, Marine Paul; CHU de Nantes, Nantes: Jean-Baptiste Lascarrou (PI), Emmanuel Canet, Charlotte Garret, Arnaud Felix Miaihle, Jean Reignier; Dupuytren Teaching Hospital, Limoges: Philippe Vignon (PI), Thomas Daix, Arnaud Desachy, Bruno Evrard, Bruno Francois, Anne-Laure Fedou, Marine Goudelin.

Germany: Charité Universitätsmedizin, Berlin: Christian Storm (PI), Gabriele Kress, Christoph Leithner, Jens Nee, Kaspar Josche Streitberger.

© 2023 American Medical Association. All rights reserved.

Italy: San Martino Policlinico Hospital, Genoa: Iole Brunetti (PI), Lorenzo Ball, Denise Battaglini, Giulia Bonatti, Iacopo Firpo, Paolo Frisoni, Arianna Iachi, Simona Maiani, Maura Mandelli, Chiara Robba, Fabio Tarantino; Civil Hospital, Baggiovara, Modena: Alberto Barbieri (PI), Elisabetta Bertellini, Enrico Giuliani, Gabriele Melegari; Trieste University hospital, Trieste: Erik Roman-Pognuz (PI), Giorgio Berlot, Umberto Lucangelo, Elisabetta Macchini.

New Zealand: Wellington Regional Hospital, Wellington: Paul Young (PI), Colin Barnes, Ben Barry, Nina Beehre, Dick Dinsdale, Sam Edney, Anna Hunt, Harriet Judd, Charlotte Latimer-Bell, Cassie Lawrence, James Moore, Shaanti Olatunji, Alex Psirides, Chelsea Robinson, Kate Tietjens, Jason Wright; Christchurch Hospital, Christchurch: David Knight (PI), Brandon Birker, David Bowie, Tara Burke, David Closey, Rosalind Crombie, Neil Davidson, Seton Henderson, Louise Hitchings, James McKay, Jan Mehrtens, Emmeline Minto, Stacey Morgan, Anna Morris, Jay Ritzemar-Carter, Jessica Roberts, Geoffrey Shaw, Katherine Townend, Kymbalee Vander Heyden.

Norway: Oslo University Hospital, Rikshospitalet, Oslo: Jan Hovdenes (PI), Vibeke Aune, Tomas Drægni, Simon Jacobsen, Søren Pieschke, Åse Rasmussen, Gro Ringstad Akselsen; St. Olav’s University Hospital, Trondheim: Halvor Langeland (PI), Daniel Bergum, Therese M. Erbe, Pål Klepstad, Helle M. Næss; Sorlandet Hospital, Arendal: Roy Bjørkholt Olsen (PI), Lena Eriksen Skjelnes, Marius Holen, Joakim Iver Post; Haukeland University Hospital, Bergen: Rune Fanebust (PI), Linda Hårteig Sørensen, Ken Åge Kårstad, Carsten Fredrik Wickman.

Sweden: Sahlgrenska University Hospital, Gothenburg: Christian Rylander (PI), Marita Ahlqvist, Roman Desta Lindgren, Ingrid Eiving, Andreas Lundin, Patrik Martner, Elisabeth Myhrman, Birgitta Ryding; Skåne University Hospital, Malmö: Joachim Düring (PI), Mattias Bergström, Mattias Bohm, Ingrid Didriksson, Petrea Frid, Katarina Heimburg, Marina Larsson, Oscar Lundberg, Stefan Olsson Hau, Simon Schmidbauer; Skåne University Hospital, Lund: Ola Borgquist (PI), Anne Adolfsson, Anna Bjärnroos, Erik Blennow-Nordström, Irina Dragancea, Thomas Kander, Anna Lybeck, Gustav Mattiasson, Olof Persson, Malin Rundgren, Susann Schrey, Erik Westhall; Helsingborg Hospital, Helsingborg: Martin Annborn (PI), Sara Andertun, Florian Ebner, Nerida Gustavsson, Lisa Hassel, Jesper Johnsson, Marie Nelderup, Heléne Petersson, Jörgen Petersson, Frideriki Stafilidou; Hallands Hospital, Halmstad: Johan Undén (PI), Frida Antonsson, Git Bergman, Jörgen Gamroth, Maria Meirik, Katarina Rudolfsson, Helena Sandberg, Martin Thorsson; Karlstad Central Hospital, Karlstad: Kristin Savolainen (PI), Maria Hansbo, Malin Helliksson, Björne Nödtveidt, Johan Sanner, Victoria Sem, Camilla Sund Lindquist; Södersjukhuset, Karolinska Institute, Stockholm: Per Nordberg (PI), Akil Awad, Anna-Sofia Börjesson, Malin Hedberg, Mia Henning, Jacob Hollenberg; Northern Älvsborg County Hospital, Trollhättan: Per Petersen (PI),

© 2023 American Medical Association. All rights reserved.

Emelia Dahlberg, Johan Forshammar, Veronica Svensson; Capio S:t Görans Hospital, Stockholm: Michael Wanecek (PI), Håkan Eskilsson; Skaraborg Hospital, Skövde: Daniel Rodriguez-Santos (PI), Åsa Appelqvist, Henrietta Jidbratt, Elisabeth Johansson, Lars Kiszakiewicz, Åsa Nilsson, Sinnika Olsson, Anders Paulsson, Urszula Stempel, Andreas Thoren; Örebro University Hospital, Örebro: Stefan Persson (PI), Ida Berglund, Eric Bergström, Cathrine Törnqvist, Ingela Östman; Uppsala University Hospital, Uppsala: Sten Rubertsson (PI), Ing-Marie Larsson, Elin Söderman, Ewa Wallin, Joanna Wessbergh; Linköping University Hospital, Linköping: Thomas Halliday (PI), Filippa Engvall.

Switzerland: Lausanne University Hospital (CHUV), Lausanne: Mauro Oddo (PI), Nawfel Ben-Hamouda, Adriano Bernini, Pierre-Nicolas Carron, Philippe Eckert, Eva Favre, John-Paul Miroz, Paola Morelli, Olivier Muller, Jan Novi, Andrea Rosseti, Madeleine Schnorf; Bern University Hospital, Bern: Matthias Haenggi (PI), Anja Levis, Sandra Nansoz, Marianne Roth & Team, Nicole Söll; Cantonal Hospital St. Gallen, St.Gallen: Claudia Schrag (PI), Mensur Alicajic, Philipp Baier, Joel Dütschler, Dominique Flügel, Edith Fässler, Ruth Gamio-Veis, Marc Güpfert, Yvonne Hilpertshauser, Stefan Hägele-Link, Gian-Reto Kleger, Peter Krähenmann, Maria Elisabeth Mair, Nadja Schai, Christoph Strohmaier, Peter Tangl, Dominik Zieglgänsberger; University Hospital Zurich, Zurich: Marco Maggiorini (PI), Gabriele Claus, Gabi Consani-Vogel, Lukas Imbach, Samira Kaiser, Eva-Maria Kleinert, Pedro David Wendel Garcia; Cardiocentro Ticino, Lugano: Tiziano Cassina (PI), Pamela Agazzi, Bruno Capelli, Gabriele Casso, Martino Regazzi, Hervé Schlotterbeck, Gabriele Via, Michele Villa.

United Kingdom: University Hospital of Wales, Cardiff: Matt P. Wise (PI), Jenny Brooks, Eve Cocks, Jade Cole, Jacqueline Curtin, Michelle Davies, Rhys Davies, Stephen Fernandez, Julie Highfield, Helen Hill, Matt P. G. Morgan, Lydia Pennant, Sofia Rose, Emma Thomas, Angharad Williams; Royal Victoria Hospital, Belfast: Peter McGuigan (PI), Stephen Haffey, Aisling O'Neill, Kathryn Ward; Bristol Royal Infirmary, Bristol: Matthew Thomas (PI), Jeremy Bewley, Anna Chillingworth, Julie Cloake, Libby Cole, Hilary Galvin, Zoe Garland, Lisa Grimmer, Bethany Gumbrill, Lucy Howie, Rebekah Johnson, Chloe Searles, Agnieszka Skorko, Katie Sweet, Victoria Taylor, Denise Webster; Essex Cardiothoracic Centre, Basildon: Thomas Keeble (PI), Gill Adams, Rajesh K Aggarwal, Jo-Anne Cartwright, Steven Church, Gerald J Clesham, John R Davies, Kelly Farrell, Reto Gamma, Jane Harding, Rohan Jagathesan, Alamgir Kabir, Paul A Kelly, Lauren Kittridge, Maria Maccaroni, Gracie Maloney, Marco Mion, Naveen Nain, Raghunath Nalgirkar, Gyanesh Namjoshi, Stacey Pepper, Emily Redman, Nicholas M Robinson, Jeremy Sayer, Amanda Solesbury, Kare H Tang, Sali Urovi, Kunal Waghmare, Noel Watson, Teresa Webber; University Hospitals Birmingham NHS Foundation Trust, Birmingham: Peter Isherwood (PI), Conor Bentley, Colin Bergin, Ronald Carrera, Amy Clark, Lauren Cooper, Liesl Despy, Natalie Dooley,

© 2023 American Medical Association. All rights reserved.

Karen Ellis, Emma Fellows, Stephanie Goundry, Samantha Harkett, Christopher McGhee, Aoife Neal, Hazel Smith, Catherine Snelson, Elaine Spruce, Tony Whitehouse, Kamal Yakoub; Royal Berkshire Hospital, Reading: Andrew Walden (PI), Shauna Bartley, Parminder Bhuie, Matthew Frise, Nicola Jacques, Liza Keating; Queen Alexandra Hospital, Portsmouth: David Pogson (PI), Zoe Daly, Steve Rose; Manchester Royal Infirmary, Manchester: Jonathan Bannard-Smith (PI), Rachael Quayle; Royal Bournemouth Hospital, Bournemouth: Nigel Chee (PI), Nina Barratt, Katie Bowman, Debbie Branney, Elizabeth Howe, Maria Letts, Sally Pitts, Luke Vamplew.

USA: University of Pittsburgh, Pittsburgh PA: Clifton W. Callaway (PI), Sara Difiore Sprouse, Ankur A. Doshi; Mayo Clinic, Rochester MN: Jennifer Fugate (PI), Amy M. Headlee, Eelco F.M.Wijdicks.

PI – Principal Investigator

RELATED ORGANIZATIONS

Copenhagen Trial Unit, Copenhagen, Denmark; Clinical Studies Sweden – Forum South, Skåne University Hospital, Lund, Sweden; The George Institute for Global Health, Sydney, Australia; Australian and New Zealand Intensive Care Research Centre, Monash University, Melbourne, Australia; Medical Research Institute of New Zealand - (MRINZ), Wellington, New Zealand; Clinical Research Unit, Paris Descartes Necker Cochin, Paris, France; Scandinavian Critical Care Trials Group (SCCTG); The HRB Irish Critical Care- Clinical Trials Network (ICC-CTN); The ANZICS Clinical Trials Group (CTG); Spiral Software, Wellington, New Zealand; Allianz Global Corporate & Specialty SE, Copenhagen, Denmark; IBBL (Integrated BioBank of Luxembourg), Dudelange, Luxembourg. We also would like to acknowledge the CENTER-TBI project supported by the Framework 7 programme of the European Union (602150-2) for the possibility to use the translated version of the structured GOSE interview. The translations formed part of a deliverable for the Outcomes Work Package, and were the responsibility of a team at the Institute of Medical Psychology and Medical Sociology, Göttingen Medical Center (Prof. Dr. N.v.Steinbüchel, Göttingen team and external and Center-TBI collaborators). The GOSE interviews were further modified for Cardiac Arrest use by the TTM2 national investigators after approval from Prof. Lindsay Wilson.

© 2023 American Medical Association. All rights reserved.

eTable 1. Detailed description of the categories in the Glasgow Outcome Scale Extended (GOSE)

	Category	Description	Question/s and answer/s indicating category in the GOSE interview
**GOSE 1**	*Dead*	NA	NA
**GOSE 2**	*Vegetative state*	Unconscious	• Is the person able to obey simple commands, or say any words?=No. *Confirmation of vegetative state requires full assessment as in the Royal College of Physician Guidelines.*
**GOSE 3**	*Lower severe disability*	Dependent, need frequent help	• Is the assistance of another person at home essential every day for some activities of daily living?=Yes; AND, Do they need frequent help or someone to be around most of the time?=Yes
**GOSE 4**	*Upper severe disability*	Dependent, need some help	• Is the assistance of another person at home essential every day for some activities of daily living?=Yes; AND, Do they need frequent help or someone to be around most of the time?=No<br>• OR/AND, Are they able to shop without assistance?=No<br>• OR/AND, Are they able to travel locally without assistance?=No
**GOSE 5**	*Lower moderate disability*	Independent, unable to participate in one or more life roles	• Are they currently able to work to their previous capacity?=No; AND, able to work only in a sheltered workshop or non-competitive job, or currently unable to work<br>• OR/AND, Are they able to resume regular social and leisure activities outside home?=No; AND unable to participate: rarely, if ever, take part<br>• OR/AND, Have there been psychological problems which have resulted in ongoing family disruption or disruption to friendships?=Yes; AND, constant – daily and intolerable
**GOSE 6**	*Upper moderate disability*	Independent, limited to participate in one or more life roles	• Are they currently able to work to their previous capacity?=No; AND reduced working capacity<br>• OR/AND, Are they able to resume regular social and leisure activities outside home?=No; AND participate much less: less than half as often<br>• OR/AND, Have there been psychological problems which have resulted in ongoing family disruption or disruption to friendships?=Yes; AND frequent – once a week or more, but tolerable
**GOSE 7**	*Lower good recovery*	Independent, returned to normal life with some symptoms	• Are they able to resume regular social and leisure activities outside home?=No; AND participate a bit less: at least half as often as before cardiac arrest<br>• OR/AND, Have there been psychological problems which have resulted in ongoing family disruption or disruption to friendships?=Yes; AND occasional –less than weekly<br>• OR/AND, Are there any other current problems relating to the cardiac arrest which affect daily life?=Yes
**GOSE 8**	*Upper good recovery*	Independent and a full return to normal life	• Are there any other current problems relating to the cardiac arrest which affect daily life?=No

© 2023 American Medical Association. All rights reserved.

eTable 2. Occupational status at time of, and at 6-months after Out-of-hospital cardiac arrest

Occupational status	Time-point	All included		Pre-arrest workers only	
		Hypothermia	Normothermia	Hypothermia	Normothermia
No.		415	407	232	207
Working full-time[a], No. (%)	Pre-arrest	192 (46)	175 (43)	192 (83)	175 (85)
	At 180 days	88 (21)	97 (24)	87 (37)	95 (46)
Working part-time[b], No. (%)	Pre-arrest	39 (9)	32 (8)	39 (17)	32 (16)
	At 180 days	54 (13)	41 (10)	53 (23)	40 (19)
Unemployed, No. (%)	Pre-arrest	8 (2)	21 (5)	NA	NA
	At 180 days	19 (5)	22 (5)	13 (6)	6 (3)
Retired due to age, No. (%)	Pre-arrest	140 (34)	141 (35)	NA	NA
	At 180 days	149 (36)	143 (35)	9 (4)	3 (1)
Retired due to disability/health, No. (%)	Pre-arrest	20 (5)	20 (5)	NA	NA
	At 180 days	40 (10)	31 (8)	19 (8)	8 (4)
On sick leave, No. (%)	Pre-arrest	4 (1)	7 (2)	n/a	n/a
	At 180 days	52 (12)	58 (14)	47 (20)	48 (23)
Other (e.g. student, housewife/husband), No. (%)	Pre-arrest	12 (3)	11 (3)	n/a	n/a
	At 180 days	13 (3)	15 (4)	3 (1)	7 (3)

[a] Full time work defined as ≥30 hours a week
[b] Part time work defined as <30 hours a week

© 2023 American Medical Association. All rights reserved.

eTable 3. Results of individual items of the Montreal Cognitive Assessment (MoCA)

**Cognitive domains**		**MoCA-30[a]**		**MoCA**		**T-MoCA**	
No.		760		607		153	
MoCA item	Maximum item score	Raw scores Mean (SD)	Percent of maximum score	Raw scores Mean (SD)	Percent of maximum score	Raw scores Mean (SD)	Percent of maximum score
Visuoexecutive	5 points	4.01 (1.26)	80%	4.01 (1.26)	80%	n/a	n/a
Naming	3 points	2.88 (0.46)	96%	2.88 (0.46)	96%	n/a	n/a
Attention-Digit span	2 points	1.75 (0.51)	88%	1.75 (0.51)	88%	1.76 (0.51)	88%
Attention- Letters	1 point	0.93 (0.26)	93%	0.94 (0.25)	94%	0.91 (0.29)	91%
Attention-Calculation	3 points	2.66 (0.77)	89%	2.69 (0.73)	90%	2.56 (0.90)	85%
Repetition	2 points	1.62 (0.66)	81%	1.64 (0.64)	82%	1.54 (0.73)	77%
Verbal fluency	1 point	0.59 (0.49)	**59%**	0.57 (0.50)	**57%**	0.65 (0.48)	**65%**
Abstraction	2 points	1.64 (0.62)	82%	1.62 (0.63)	81%	1.71 (0.58)	86%
Delayed recall	5 points	2.95 (1.72)	**59%**	2.88 (1.74)	**58%**	3.21 (1.60)	**64%**
Orientation	6 points	5.67 (0.97)	94%	5.70 (0.89)	95%	5.52 (1.21)	92%

The two items with the most participants having low scores (less percent of maximum) indicated with bold text.

[a] including converted T-MoCA

Abbreviations: MoCA=Montreal Cognitive Assessment; T-MoCA=Telephone version of the Montreal Cognitive Assessment

© 2023 American Medical Association. All rights reserved.

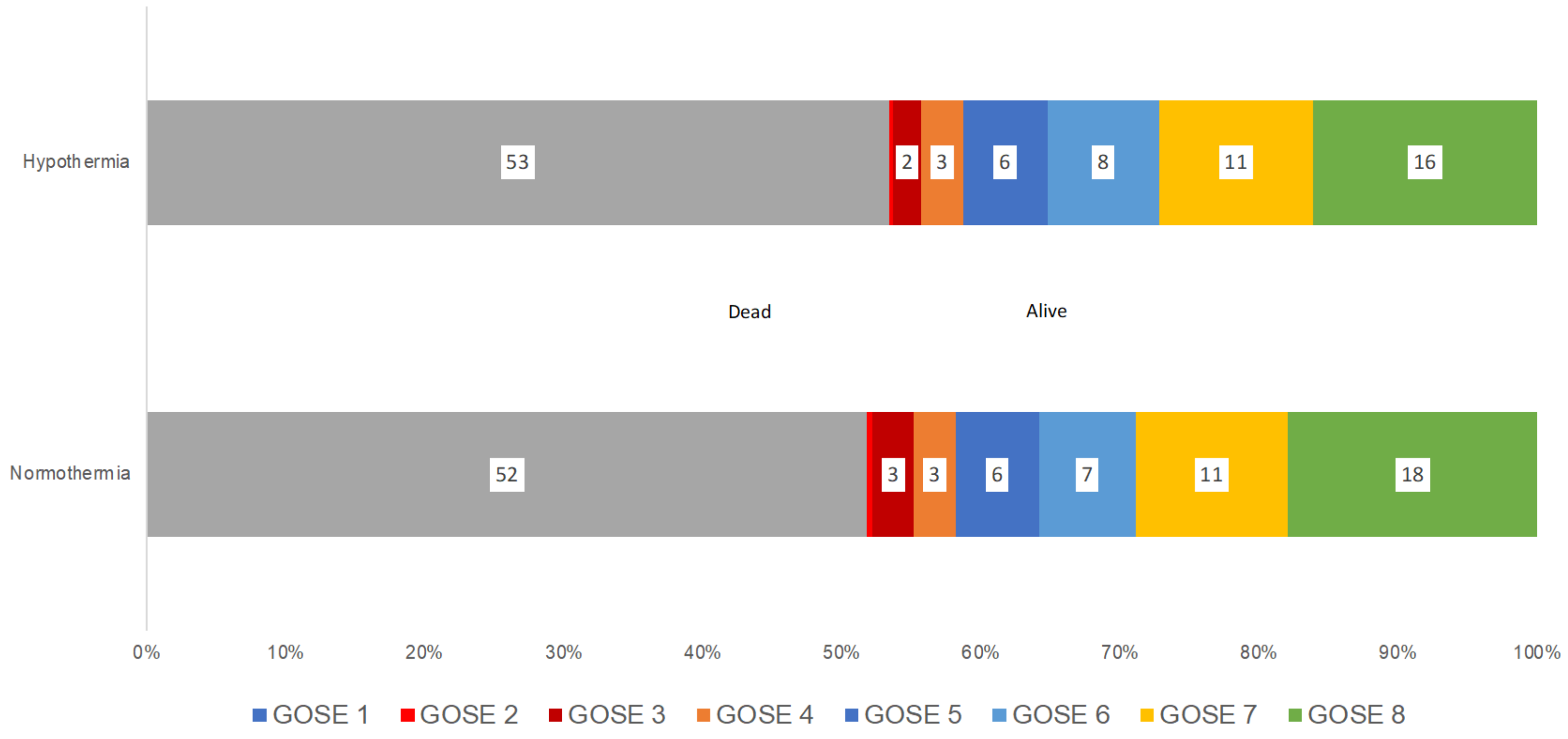

eFigure 1*:* Functional outcome focusing on societal all participants including death

The Glasgow Outcome Scale Extended (GOSE) for participants with hypothermia (n=880) and normothermia (n=865) at 6-months after out-of-hospital cardiac arrest. *Categories: GOSE 1 dead*; *GOSE 2 vegetative state* (unconscious); *GOSE 3 lower severe disability* (dependent, need frequent help); *GOSE 4 upper severe disability* (dependent, need some help); *GOSE* 5 lower moderate disability (independent, unable to participate in one or more life roles), *6 upper moderate disability* (independent, limited to participate in one or more life roles)*, 7 lower good recovery* (independent, returned to normal life with some symptoms); and *GOSE 8 upper good recovery* (independent and a full return to normal life).

© 2023 American Medical Association. All rights reserved.

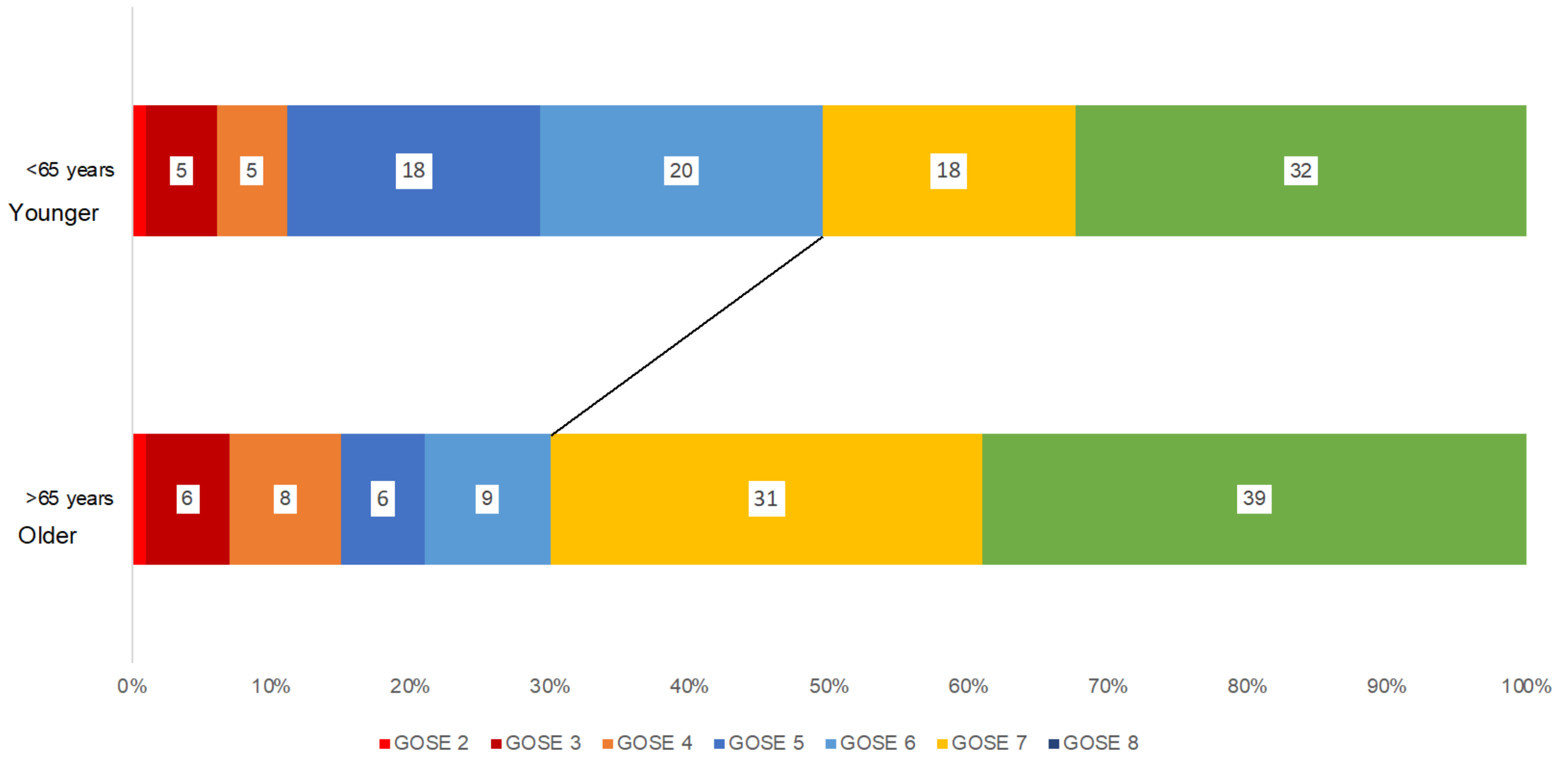

eFigure 2: Functional outcome focusing on societal participation by age groups

The Glasgow Outcome Scale Extended (GOSE) for younger (n=494) and older (n=340) survivors (n=834) at 6-months after out-of-hospital cardiac arrest. *Categories: GOSE 2 vegetative state* (unconscious); *GOSE 3 lower severe disability* (dependent, need frequent help); *GOSE 4 upper severe disability* (dependent, need some help); *GOSE* 5 lower moderate disability (independent, unable to participate in one or more life roles), *6 upper moderate disability* (independent, limited to participate in one or more life roles*), 7 lower good recovery* (independent, returned to normal life with some symptoms); and *GOSE 8 upper good recovery* (independent and a full return to normal life).

© 2023 American Medical Association. All rights reserved.

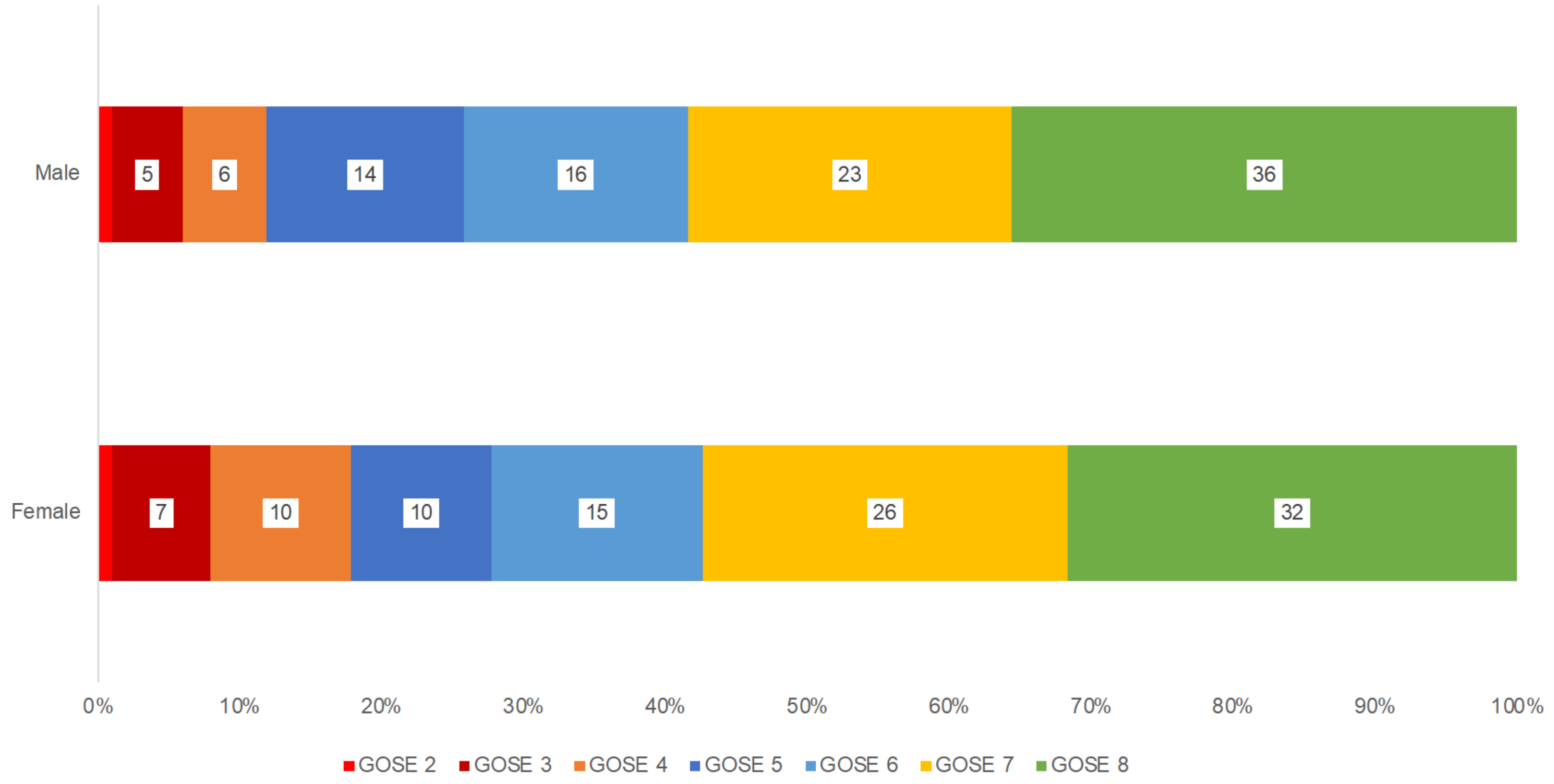

eFigure 3: Functional outcome focusing on societal participation by sex

The Glasgow Outcome Scale Extended (GOSE) for survivors (n=834) at 6-months after out-of-hospital cardiac arrest for males (n=698/834) and females (n=136/834). *Categories*: *GOSE 2 vegetative state* (unconscious); *GOSE 3 lower severe disability* (dependent, need frequent help); *GOSE 4 upper severe disability* (dependent, need some help); *GOSE* 5 lower moderate disability (independent, unable to participate in one or more life roles), *6 upper moderate disability* (independent, limited to participate in one or more life roles*), 7 lower good recovery* (independent, returned to normal life with some symptoms); and *GOSE 8 upper good recovery* (independent and a full return to normal life).

© 2023 American Medical Association. All rights reserved.
