# Supplementary material for: Effects of Hypothermia vs Normothermia on Societal Participation and Cognitive Function at 6 Months in Survivors After Out-of-Hospital Cardiac Arrest: A Predefined Analysis of the TTM2 Randomized Clinical Trial
Source: JAMA Neurol. 2023 Aug 7;80(10):1070–9. doi: 10.1001/jamaneurol.2023.2536 (PMC10407762; doi:10.1001/jamaneurol.2023.2536)
Supplement: Supplement 3. — Data sharing statement [file jamaneurol-e232536-s003.pdf]

Data Sharing Statement

Lilja. The Effects of Hypothermia vs Normothermia on Societal Participation and Cognitive Function at 6 Months in Survivors After Out-of-Hospital Cardiac Arrest. *JAMA Neurol*. Published August 07, 2023. doi:10.1001/jamaneurol.2023.2536

Data
Data available: Yes
Data types: Deidentified participant data, Data dictionary
How to access data: Data are available upon resonable request to the corresponding author
When available: With publication

Supporting Documents
Document types: Other (please specify)
Additional Information: Online supplemental material Pre published protocol for the TTM2 trial and TTM2 trial follow up are published
How to access documents: Published. References are included in the mnauscript
When available: With publication

Additional Information
Who can access the data: Protocols are published and available. References are available inte the manuscript
Types of analyses: n/a
Mechanisms of data availability: n/a
Any additional restrictions: n/a
